# The Development of a Community Pharmacy-Based Intervention to Optimize Patients’ Use of and Experience with Antidepressants: A Step-by-Step Demonstration of the Intervention Mapping Process

**DOI:** 10.3390/pharmacy6020039

**Published:** 2018-05-02

**Authors:** Tania Santina, Sophie Lauzier, Hélène Gagnon, Denis Villeneuve, Jocelyne Moisan, Jean-Pierre Grégoire, Laurence Guillaumie

**Affiliations:** 1 Faculty of Nursing, Université Laval, Pavillon Ferdinand-Vandry, 1050, Avenue de la Médecine, Quebec City, QC G1V 0A6, Canada; tanya.santina.1@ulaval.ca; 2 Faculty of Pharmacy and Centre de Recherche du CHU de Québec-Université Laval, Hôpital Saint-Sacrement, 1050 Chemin Ste-Foy, Quebec City, QC G1S 4L8, Canada; sophie.lauzier@pha.ulaval.ca (S.L); jocelyne.moisan@pha.ulaval.ca (J.M.); jean_pierre.gregoire@pha.ulaval.ca (J.-P.G.); 3 Quebec City, QC G3B 1Y5, Canada; helene.z.gagnon@gmail.com; 4 Panacée Conseil, Quebec City, QC G2J 0A2, Canada; denis.villeneuve@panacee.ca

**Keywords:** Intervention Mapping, program development, antidepressant drugs, anxiety disorder, mood disorder, community pharmacy services, patient education, patient satisfaction, medication adherence

## Abstract

**Objective:** To describe the development of a community pharmacy-based intervention aimed at optimizing experience and use of antidepressants (ADs) for patients with mood and anxiety disorders. **Methods:** Intervention Mapping (IM) was used for conducting needs assessment, formulating intervention objectives, selecting change methods and practical applications, designing the intervention, and planning intervention implementation. IM is based on a qualitative participatory approach and each step of the intervention development process was conducted through consultations with a pharmacists’ committee. **Results:** A needs assessment was informed by qualitative and quantitative studies conducted with leaders, pharmacists, and patients. Intervention objectives and change methods were selected to target factors influencing patients’ experience with and use of ADs. The intervention includes four brief consultations between the pharmacist and the patient: (1) provision of information (first AD claim); (2) management of side effects (15 days after first claim); (3) monitoring treatment efficacy (30-day renewal); (4) assessment of treatment persistence (2-month renewal, repeated every 6 months). A detailed implementation plan was also developed. **Conclusion:** IM provided a systematic and rigorous approach to the development of an intervention directly tied to empirical data on patients’ and pharmacists’ experiences and recommendations. The thorough description of this intervention may facilitate the development of new pharmacy-based interventions or the adaptation of this intervention to other illnesses and settings.

## 1. Introduction

Mood and Anxiety Disorders (MADs) are the most prevalent mental illnesses in Canada [1]. In 2013, 11.6% of Canadians adults reported having a MAD [2]. MADs have been shown to be associated with chronic illnesses such as respiratory and heart diseases [1]. They also have negative consequences on patients’ social relationships and quality of life, increase the risk of suicide, and represent an important economic burden to society [3,4,5]. Antidepressants (ADs) are recommended by the Canadian Network for Mood and Anxiety Treatments (CANMAT) for the treatment of MADs, alone or in conjunction with psychotherapy [3,5]. According to the CANMAT guidelines, ADs should be continued for several weeks after full response: 6–24 weeks for depression [3] and 12–24 weeks for anxiety disorders [5,6]. MAD patients report several needs regarding ADs, and a high proportion of patients will end treatment prematurely [7,8,9,10,11].

Several studies have explored factors that negatively influence patients’ experiences with and adherence to ADs. A meta-ethnography conducted among patients with depression showed that patients constantly re-evaluate the relevance of antidepressant drug treatment and reassess their willingness to continue treatment [12]. Adverse effects [13,14], lack of support from the prescribing physician [14,15], lack of confidence in the efficacy of ADs [16], holding a negative opinion of ADs [12,13,17,18], and being strongly affected by the social stigma associated with the diagnosis of a mental health problem [16,19] appeared to negatively influence AD adherence and patients’ experiences with treatment. In a literature review [11], poor instruction about ADs, lack of follow-up from the prescribing clinician, patients’ fear of addiction, low motivation, lower depression severity, and a complex drug regimen were also associated with nonadherence in some studies.

Community pharmacists can play a pivotal role in addressing issues faced by patients prescribed ADs for MADs [20]. Community pharmacists have frequent interactions with these patients and some previous community pharmacy-based interventions conducted among patients with depression [21] or common mental illnesses (primarily anxiety or depression) [22] reported promising results for improving patients’ adherence to ADs. Findings from a systematic review [21] and meta-analyses of controlled trials reported significant effects on AD adherence (odds ratio of 2.5) for interventions delivered by pharmacists in outpatient clinics or community pharmacies but reported non-significant effects on the reduction of clinical symptoms [23]. Some of these reviews concluded that better results could be expected for adherence and reduction of clinical symptoms in better-designed interventions. Indeed, although interventions included patient education [22,24,25,26,27,28,29,30,31], monitoring symptoms [24,25,26,27,28,29], and management of side effects [27,32], they mainly targeted one phase of AD adherence (initiation or maintenance) and most studies failed to provide a detailed description of the intervention content or the anticipated change process and did not control for the extent to which the intervention was implemented. All of these factors may have lowered the potential effects of the intervention. In addition, to the best of our knowledge, only one of these interventions was designed using behavior change theories and a structured approach to intervention development [22].

Intervention Mapping (IM) [33] is a step-by-step protocol that assists planners in developing complex health promotion interventions [34]. Each step of the intervention development is based on a qualitative participatory approach involving consultations with relevant stakeholders to elucidate the challenges and facilitating factors that may affect the success of the intervention [33]. Previous reviews of controlled studies report that interventions based on IM showed significant results on health promotion behaviors [35] and on the adoption of innovative health care practices [36]. More specifically, earlier studies have demonstrated that interventions based on IM significantly improved medication adherence for antiretroviral therapy [37] and drug treatment for depression and anxiety [22], among others. This highlights the relevance of using the IM process for designing interventions with potential for efficacy [33,38].

The aim of the present paper is to present the Intervention Mapping process that was followed for the development of a community pharmacy-based intervention aimed at optimizing the use of ADs for patients with MADs as well as their experience with the treatment.

## 2. Methods

### 2.1. Study Design and Setting

The current study was carried out in the province of Quebec, Canada. As recommended by the developers of the IM protocol [33], a qualitative participatory approach was used to construct and develop the intervention [39]. This involved collaboration between researchers and community pharmacists in order to share knowledge and develop actions that take into account the perspectives of different actors, and it took the form of a pharmacists’ committee that was involved in each step of the process. This committee consisted of four community pharmacists. The size of this group was judged appropriate to facilitate discussions as, prior to meetings with this pharmacists’ committee, we also gained important input on the role of community pharmacists for patients taking ADs by conducting one quantitative and three qualitative studies [40,41,42,43]. Committee members were identified through the contacts of the research group. To be included in the committee, members had to (1) be currently working as a community pharmacist, or to have worked as a community pharmacist and be currently involved in training pharmacy students; and (2) be available for and willing to participate in the intervention development meetings. The pharmacists’ committee included two men and two women. Committee members were all more than 40 years old and had extensive experience in community pharmacy (with pharmacy degrees obtained more than 15 years ago) as owners or salaried employees. Some of them knew each other prior to the establishment of the committee. Only one member reported significant previous experience in developing an intervention to improve medication adherence. As committee members were more familiar with the practices of community pharmacists than the research and intervention development processes, they were very conscious of the feasibility and the operational aspects of the intervention under development. Meetings with the committee were conducted to develop an understanding of pharmacists’ points of view, provide opportunities for sharing experiences, and to make decisions and integrate these decisions into a concrete intervention plan. The committee was facilitated by one expert in IM (HG) and two researchers (patient health education, LG; epidemiology, SL). One researcher (LG) was trained in cognitive behavioral approaches and more specifically in behavior change theories. One researcher (SL) was trained in social and cultural anthropology and epidemiology with an expertise in medication use research. This influenced the decisions made throughout the intervention development process, such as the selection of theoretical methods and practical applications as well as the concrete development of the intervention design. The facilitators’ roles were to provide the documentation necessary to support the reflective process prior to meetings and to facilitate the meeting discussions. Concretely, each meeting was devoted to one of the six steps of the IM protocol. At the beginning of each meeting, the facilitators presented the tasks associated with the step and provided the documentation needed to carry out the tasks. Occasionally, the facilitators supplied suggestions or a first draft as a basis for discussion. The number of meetings was not defined a priori and was dependent on the time necessary to complete the development of the intervention.

### 2.2. The Intervention Mapping Protocol for Designing Interventions

**Figure 1:**
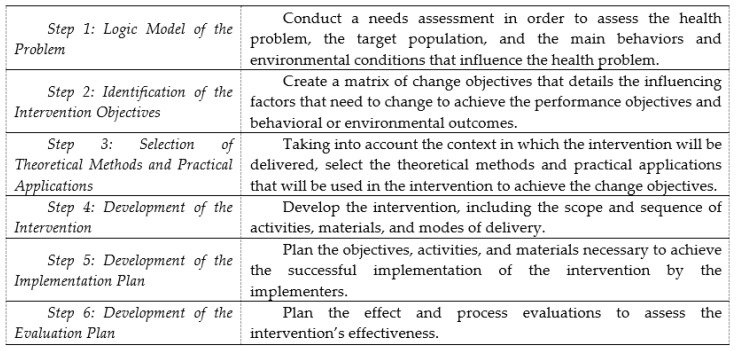
Intervention Mapping protocol steps.

The step-by-step process of IM allows the development of health promotion interventions based on theories, scientific literature, and data collected in the field [33,38]. It comprises six fundamental steps that build on each other. Although IM is presented as a series of steps, the planning process is iterative rather than linear [33,38]. Each step is conducted in partnership with a steering committee composed of relevant stakeholders. The use of a participatory approach for the development of the intervention is at the core of the Intervention Mapping process. Figure 1 depicts these steps.

*Step 1: Logic Model of the Problem.* In IM [33], the development of an intervention should be preceded by a needs assessment in order to assess the health problem, the target population, the main behaviors and environmental conditions that influence the health problem, the main factors that influence these behaviors and environmental conditions, and the characteristics of previous interventions for a similar issue. This involves an extensive review of the literature and primary research.

*Step 2: Identification of the Intervention Objectives.* At this step, measurable behavioral and environmental outcomes are formulated. For some interventions, only behavioral or environmental outcomes may be relevant, while for other interventions, both are important. In the present study, only one behavioral outcome was formulated (e.g., each adult with a new prescription has an optimal experience with and use of ADs) since the achievement of this behavioral outcome was expected to improve the targeted health outcomes. Then, each *behavioral outcome* is subdivided into *performance objectives*, which are the logical and procedural steps necessary for the individual to achieve the behavioral outcomes [33] (e.g., the patient verbally commits to a systematic follow-up plan with the pharmacist). On the basis of the needs assessment, the factors influencing each behavioral outcome are linked to relevant performance objectives in a table, thereby creating a matrix of *change objectives* (e.g., the patient knows that he/she can contact a pharmacist) that details how these influencing factors need to change to achieve the performance objectives and behavioral outcomes.

*Step 3: Selection of Theoretical Methods and Practical Applications*. To operationalize the change objectives into practical applications that will be used in the concrete intervention, theoretically informed methods are selected, taking into account the context and environment in which the intervention will be delivered. The selection is based on a taxonomy developed by the originators of IM [33], the empirical effectiveness and feasibility of these methods for achieving the behavioral outcomes, and performance objectives in similar populations and settings [44,45,46]. One class of methods in this taxonomy is the “basic methods”, which are likely to influence several factors related to behavioral adoption. Other classes of behavior change methods are specific to a factor influencing behavior adoption (e.g., self-efficacy) and a theory (e.g., social cognitive theory).

*Step 4: Development of the Intervention*. This step involves the development of the intervention itself, including the scope and sequence of activities, materials, and modes of delivery. The intervention content and materials are determined in relation to the change objectives formulated in Step 2 and the theoretical methods and practical applications selected in Step 3. Finally, the whole intervention is verified to ensure that it meets the characteristics defined in Steps 1, 2, and 3.

*Step 5: Development of the Implementation Plan.* In a similar manner to what is described in Step 2, a matrix is elaborated for each behavioral outcome (e.g., drug therapy monitoring is systematically implemented) that describes what is needed to achieve the successful implementation of the intervention by the implementers (defined as those with a role in the implementation process). The influencing factors of each behavioral outcome (e.g., knowledge, self-identity) are identified based on the findings of the needs assessment and are linked to performance objectives to create a matrix of change objectives [33]. The change objectives are converted into practical applications based on a range of evidence and the taxonomy developed by Kok et al. [33,38].

*Step 6: Development of the Evaluation Plan.* Evaluations to assess the effects and processes of the intervention are designed by selecting evaluation objectives and deciding on indicators, their measures, and data collection procedures. Mixed research methods are usually involved in evaluation planning.

## 3. Results

### 3.1. Step 1: Logic Model of the Problem

A needs assessment was conducted that included literature reviews on several topics: (1) patients’ use of ADs and associated factors; (2) patients’ experience with ADs and associated factors; and (3) the characteristics, effects, and limits of community pharmacy-based interventions that target patients’ use of and experience with ADs. While patients’ experiences with ADs and the challenges patients face have been extensively documented in the published literature, there was a lack of information on patients’ experiences of pharmacy services and the actual and optimal pharmacy practices for this population. To complete the information collected from these reviews, three descriptive exploratory qualitative studies were conducted: (1) individual interviews with patients prescribed ADs (*n* = 14) [40]; (2) individual interviews with key informants in mental health and pharmacist practices (*n* = 21) [41]; and (3) focus groups with community pharmacists (*n* = 43) [42]. The aims of these studies were the following: (1) to describe community pharmacists’ current practices and challenges; (2) to explore the factors influencing the initiation of ADs and persistence for the whole length of treatment; and (3) the potential contributions of community pharmacists to improve patients’ use of and experience with ADs. Following these qualitative studies, a cross-sectional study was conducted among community pharmacists in the province of Quebec (*n* = 1609) to identify the psychosocial factors influencing whether pharmacists would deliver four interventions per year to enhance patients’ use of and experience with ADs [43]. The key findings of these studies are presented in Table 1. Findings from this needs assessment were presented and discussed at the first meeting with the pharmacists’ committee.

### 3.2. Step 2: Identification of the Intervention Objectives

The intervention objectives were formulated collaboratively with the pharmacists’ committee during the second meeting. The behavioral objective of the intervention was “each adult with a MAD who presents with a new prescription for ADs at pharmacy has an optimal experience with and use of ADs.” Six performance objectives were formulated in the following order: (PO1) the patient verbally commits to a systematic pharmaceutical follow-up plan with the pharmacist that includes at least four brief consultations; (PO2) the patient makes an informed decision to initiate ADs; (PO3) the patient takes the ADs as prescribed throughout the treatment period (dosage, time, and frequency); (PO4) the patient copes with the side effects of the treatment; (PO5) the patient assesses the benefits of taking the ADs; and (PO6) the patient makes an informed decision to persist with the treatment throughout the length of the prescription. Change objectives were formulated by crossing performance objectives to the influencing factors identified in Step 1: knowledge, attitude, self-efficacy, and intention (Table 2).

### 3.3. Step 3: Selection of Theoretical Methods and Practical Applications

In a third meeting with the pharmacists’ committee, theoretical methods and practical applications to change the influencing factors specified in Step 2 were organized into a coherent intervention. Participation, discussion, individualization, belief selection, reinforcement, and anticipation of the adaptation strategies to be employed were chosen (see Kok’s taxonomy for the definitions and associated practical applications) [38]. For example, reinforcement, derived from social cognitive theory [47] was translated into a practical application by pharmacists providing encouragement and rewards to patients. All the selected methods belong to the category of “basic methods” that are defined in IM as being useful for several individual influencing factors, including those identified in our study (e.g., participation may be useful in modifying knowledge, attitude and self-efficacy). The authors of the IM protocol recommend prioritizing these methods as their efficacy has been empirically and extensively demonstrated in interventions at the individual level. In addition, these basic methods were deemed to be the most promising in a context where the intervention is very brief and as likely to increase pharmacists’ adoption of the intervention. The full list of the selected theory-based methods and their translation into a range of practical intervention applications is presented in Table 3.

### 3.4. Step 4: Development of the Intervention Design

In a fourth meeting with the pharmacists’ committee, the intervention components were selected based on the needs assessment (see details in Step 1). The intervention components were identified based on the change objectives (Step 2). The resultant intervention was to consist of four patient consultations of 3–5 min each: (1) providing information (at initial ADs claim); (2) management of side effects (15 days after first claim); (3) monitoring treatment efficacy (at 30-day renewal); and (4) assessment of treatment persistence (at 2-month renewal). This fourth consultation was to be repeated every 6 months or as needed. The theoretical methods selected in Step 3 (participation, discussion, individualization, belief selection, reinforcement and anticipation of the adaptation strategies to be employed) and their associated practical applications are to be used concomitantly in each of these patient consultations (and not in one patient consultation in particular). These four patient consultations are to be supported by a brief written document that lists the essential information to discuss with the patient, can be used by the pharmacist during a consultation, and can be given to the patient (this document is currently in development). Some patients who discontinue treatment without informing the pharmacist would be identified at a subsequent visit through the pharmacy’s computer system (whatever the medication requested at that visit). However, no proactive procedure such as a telephone follow-up was planned. This was mainly because such follow-up could not realistically be carried out as part of pharmacists’ usual practices. Detailed information on the sequential components of the intervention is provided in Table 4.

### 3.5. Step 5: Development of the Adoption and Implementation Plan

To ensure the implementation of the intervention in community pharmacies, a second matrix targeting community pharmacists was developed. Three performance objectives were identified: (1) pharmacists become familiar with the content of the four consultations comprising the intervention and adopt this systematic drug therapy monitoring intervention; (2) pharmacists make adjustments to their environment to facilitate the implementation of the intervention; (3) the pharmacists and the pharmacy team decide on a specific date for initiating the intervention. Change objectives were formulated by crossing the performance objectives to the factors influencing pharmacists’ intention to provide four consultations monitoring patients’ use of and experience with ADs identified in our cross-sectional study [43]. Detailed information on this matrix of objectives and the associated theoretical methods and applications is provided in Table 5 and Table 6.

### 3.6. Step 6: Development of the Evaluation Plan

The research protocol for evaluating the processes and effects of the intervention is currently under development. Objectives will be selected from those formulated (health and behavioral outcomes, performance and change objectives) to guide the effect evaluation; the process evaluation will be based on the parameters for use and the quality of the implementation. A pilot study should first be conducted to refine the intervention before conducting a large-scale study.

## 4. Discussion

This systematic process based on the IM protocol resulted in a comprehensive intervention built in partnership with community pharmacists; it was based on theoretical models, best available scientific evidence, and empirical data collected among the targeted populations (e.g., patients, community pharmacists, and leaders in pharmacy and mental health). This process is likely to increase the potential efficacy of the intervention thus developed and improve its implementation. This study is one of the few to offer a detailed description of the development process and the theoretical underpinnings of a pharmacy-based intervention designed to improve the use and experience of ADs for patients with MADs. Except for two interventions that were also modeled on IM [55,56], the vast majority of previous community pharmacy-based interventions intending to optimize experience and/or use of drug treatments for patients with mental illnesses did not seem to have been developed using a structured approach in terms of intervention development and behavior change theories. 

During the development process, several benefits to using IM were observed. First, IM provided a systematic step-by-step approach to develop the intervention. IM offered a clear set of tasks to sequentially guide and focus the meetings with the pharmacists’ committee through different questions and decisions regarding the intervention development [34]. Second, IM provided a robust methodology to concretely integrate the results of the original qualitative and quantitative studies, carried out as needs assessment, into the intervention development process [40,41,42,43]. The results from these studies provided the researchers and pharmacists’ committee with a deep understanding of the challenges and incentives that may influence both pharmacists’ practices and patients’ experience with and use of ADs. It should be acknowledged that such extensive preliminary work is not always performed prior to intervention development meetings. In such cases, a greater number of participants and committee meetings are recommended for the development of the intervention. Third, the IM approach provided a template for reflecting and deciding on the extent and the exact modalities of community pharmacists’ involvement in the intervention [36]. IM guided the identification of key leverage points and provided useful checks and balances throughout the intervention development process [57]. The intent was to improve the effectiveness and relevance of the intervention from the perspective of patients, community pharmacists, and the population of Quebec [33]. Fourth, IM enabled the thorough description of this pharmacy-based intervention and of the rationale underlying the decisions. Such descriptions will facilitate the replication and analysis of this intervention in future studies and reviews [58]. It may also support the development of new pharmacy-based interventions or the adaptation of this intervention to other illnesses and settings.

Nevertheless, the limitations related to the use of the IM process should be highlighted. Mainly, the needs assessment and the intervention development process were time-consuming. This challenge has been reported in a previous study that used IM to improve medication adherence [57]. Since researchers, patients, pharmacists, and leaders in pharmacy and mental health may hold different perspectives, a significant amount of work was necessary to incorporate these different views and priorities into a concrete intervention. In addition, the iterative process inherent in intervention development may lead to multiple revisions of the intervention prior to obtaining a consensual version.

## 5. Conclusions

This paper described the systematic development of a community pharmacy-based intervention aimed at optimizing the use of and experience with ADs for patients with MADs. It was based on the IM protocol, which involves a step-by-step process and a qualitative participatory approach. Through this approach, IM offered a transparent, problem-solving procedure to address the needs of patients prescribed ADs and the challenges of community pharmacists’ practice; it makes use of theory, research evidence, and the perspectives of patients, community pharmacists, and leaders in pharmacy and mental health. The next phases of the research will involve conducting a pilot study to assess the feasibility, acceptability, and preliminary effects of this intervention and a larger-scale study to evaluate the processes and impacts of the intervention. The planning process used was based on a robust methodology and resulted in a thorough description of the pharmacy-based intervention. This should facilitate its evaluation, replication, and adaptation to other illnesses or settings.

## Figures and Tables

**Table 1:** Synthesis of four studies conducted to assess patients’ and community pharmacists’ needs regarding antidepressant (AD) treatment (Step 1 of the Intervention Mapping protocol).

Perspective	Health Care Leaders	Patients Taking ADs	Community Pharmacists (I)	Community Pharmacists (II)
Study Objective	To explore the perspectives of leaders in pharmacy and mental health on the current and potential contributions of community pharmacists for patients on ADs [41].	To explore patients’ experiences with community pharmacy services for ADs and avenues for improvement [40].	To describe community pharmacists’ perceptions of their practices around patients with a prescription for ADs [42].	To identify factors from the theory of planned behavior associated with community pharmacists’ intention to perform systematic AD monitoring * [43].
Population	Leaders in health care, pharmaceutical services, physician and pharmacist education, and patient and healthcare professional associations.	Patients diagnosed with major depression who were prescribed ADs.	Community pharmacists in 5 regions of the province of Quebec.	Community pharmacists in the province of Quebec.
Design	Qualitative descriptive exploratory study.	Qualitative descriptive exploratory study.	Qualitative descriptive exploratory study.	Cross-sectional population-based study.
Methods	21 interviews with leaders	Individual interviews with 14 patients.	6 focus groups with 43 pharmacists	Questionnaire completed by 1609 community pharmacists.
Key Results	Pharmacists were perceived as accessible drug experts whose particular strengths are the following: (1) thorough knowledge of drugs; (2) commitment to ensure safety and tolerability; (3) commitment to inform and support patients. Leaders perceived the need for enhanced pharmacist monitoring of AD adherence and efficacy. Leaders stated that health care teams could also benefit from pharmacists’ expertise.	Patients reported that pharmacists concentrate their involvement at initiation and the first refill and that pharmacists’ contributions mainly consisted of providing information and reassurance. Patients’ expectations were that pharmacists: (1) extend their involvement by providing information throughout the length of treatment; (2) enhance the confidentiality of discussions in pharmacy.	Major aspects of current pharmacist practice around ADs: (1) convincing patients to initiate ADs; (2) dealing with side effects in the first weeks of treatment; (3) intervening mainly when patients have questions for the remainder of treatment. Challenges were mainly organizational (e.g., lack of time and remuneration). Recommendations to improve practice: (1) clear guidelines for monitoring patients; (2) better training for pharmacy technicians; (3) providing educational tools to the patient; (4) improving pharmacy software to facilitate monitoring.	Systematic AD monitoring has not been widely adopted by pharmacists, and pharmacists’ intention to perform systematic AD monitoring is moderate. Psychosocial factors associated with the intention to perform systematic AD monitoring include attitude, perception of control, subjective norms, and professional identity.

* Systematic ADs monitoring was defined as performing four consultations with each patient treated for depression during the first year of AD treatment to address side effects, treatment efficacy, and adherence.

**Table 2:** Matrix of objectives (Step 2 of the Intervention Mapping protocol). Behavioral outcome: Each adult with a Mood and Anxiety Disorder (MAD) presenting with a new prescription for ADs at pharmacy has an optimal experience with and use of ADs.

Performance Objectives	Influencing Factors			
	Knowledge	Attitude	Self-Efficacy	Intention
PO1. The patient verbally commits to a systematic pharmaceutical follow-up plan with the pharmacist that includes at least four brief consultations.	K1. The patient knows that he/she can contact a pharmacist if he/she has any questions or difficulties throughout the whole length of treatment.	A1. The patient recognizes the benefits of consulting with a pharmacist at different points during treatment.		
PO2. The patient makes an informed decision to initiate ADs.	K2. The patient knows the different phases of treatment (acute, maintenance, cessation).	A2. The patient has realistic expectations about the benefits of the ADs.		I1. The patient expresses a positive intention to initiate treatment.
	K3. The patient knows the general mechanism of action of the ADs.			
	K4. The patient knows the non-pharmacological measures that may be used in addition to ADs.			
	K5. The patient knows the potential benefits of ADs and when they may occur.			
	K6. The patient identifies the symptoms that affect him/her the most and those for which he/she expects to see positive effects.			
	K7. The patient knows the possible side effects of ADs and their evolution over time.			
PO3. The patient takes the ADs as prescribed throughout the treatment period (dosage, time, and frequency).	K8. The patient knows how to take the drug (timing, dosage, missed doses, contraindications).	A3. The patient recognizes the benefits of taking the ADs as prescribed throughout the treatment period.	SE1. The patient identifies the barriers that may hinder him/her from taking the ADs as prescribed throughout the treatment period.	
			SE2. The patient identifies strategies to overcome these barriers and makes use of them.	
PO4. The patient copes with the side effects of the treatment.	K9. The patient identifies the side effects that he/she experiences as a result of the ADs.		SE3. The patient identifies strategies to overcome these side effects and makes use of them.	I2. The patient expresses his/her intention to continue the treatment despite side effects.
PO5. The patient assesses the benefits of taking the ADs.		A4. The patient perceives the benefits of the treatment despite the presence of side effects.		
		A5. The patient recognizes that his/her main symptoms are resolved or are in the process of being resolved.		
PO6. The patient makes an informed decision to persist with the treatment throughout the length of the prescription.	K10. The patient knows the potential risks associated with premature discontinuation of the treatment.	A6. The patient recognizes the benefits of continuing the treatment for the prescribed period.	SE4. The patient identifies barriers that may hinder him/her from continuing treatment for the prescribed period.	I3. The patient expresses a positive intention to continue treatment even if the main symptoms have resolved.
			SE5. The patient identifies strategies to overcome these barriers and makes use of them.	

**Table 3:** Theoretical methods, application parameters and practical applications (Step 3 of the Intervention Mapping protocol).

Methods (Related Theory)	Definition	Parameters	Practical Applications
Participation (Motivational Interviewing) [48]	Ensuring a high degree of patient engagement in decision making, treatment taking, and problem solving.	The health care provider accepts that the patient influences the content of their encounter and that the patient requires support in terms of enhancing motivation and developing appropriate skills.	Ask about the expected benefits, side effects, perceived benefits, and intent to initiate and persist with the treatment.
			Discuss problem-solving strategies.
			With the patient, identify difficulties encountered and ways of dealing with them.
Discussion (Elaboration Likelihood Model of Persuasion) [49]	Encourage the exploration of topics in open and informal debate.	Listen to the patient and ensure that beliefs conducive to the adoption of the health behavior are activated.	Ask about the expected benefits, side effects, perceived benefits, and intent to initiate and persist with the treatment.
			Discuss problem-solving strategies.
			With the patient, identify difficulties encountered and ways of dealing with them.
Individualization (Transtheoretical Model) [50]	Provide the opportunity for patients to receive answers to their personal questions or information based on their own experience.	Communication from the health care provider is personalized and responds to the specific needs of the patient.	Provide personalized information (depending on the clinical or experiential characteristics of the patient).
			Ask about the expected benefits, side effects, perceived benefits, and intent to initiate and persist with the treatment.
			Discuss problem-solving strategies.
			Reward, praise efforts or progress, focus on successes.
			With the patient, identify difficulties encountered and ways of dealing with them.
Belief Selection (Theory of Planned Behavior) [51]	Use messages that reinforce positive beliefs, diminish negative beliefs, and introduce new beliefs.	Attitudinal, normative, and control beliefs targeted by the health care professional must have been previously documented.	Provide general information about the disease and treatment (benefits, disadvantages).
			Provide personalized information (depending on the clinical or experiential characteristics of the patient).
Reinforcement (Social Cognitive Theory) [52]	Reinforce patient’s actions or comments that may increase the likelihood of adopting the targeted behavior or its frequency.	Reinforcement must be personalized and should follow an action or statement made by the patient. Reinforcement must be seen as a consequence of the patient’s action or statement.	Reward, praise efforts or progress, focus on successes.
Anticipation of the Adaptation Strategies to be Employed (Relapse Prevention Theory) [53]	Lead the patient to identify potential barriers and ways to overcome them.	Identify risk situations and adaptation strategies.	Discuss problem-solving strategies.
			With the patient, identify difficulties encountered and ways of dealing with them.

**Table 4:** Sequence, content, objectives and documents used (Step 4 of the Intervention Mapping protocol).

Brief Consultations with the Patient	Information to Be Transmitted or Discussed with the Patient	Information to Be Obtained	Change Objectives Targeted	Documents Used
Providing information (at initial AD claim).	Disease, mechanism of action of the ADs, treatment phases, onset of treatment efficacy, possible side effects, complementary non-pharmacological measures for treatment. Directions for drug intake. Concepts of treatment compliance and treatment persistence and their importance. Both pharmacist (on behalf of the pharmacists team) and the patient commit to treatment follow-up.	Reason for prescription; confirm whether this is the patient’s first AD prescription. Patient’s therapeutic goals: identify 2 symptoms for which the patient wishes to see improvement. Inquire about the patient’s main concerns. Confirm intention to start treatment. Verbal agreement to a follow-up in about 15 days.	C1, C2, C3, C4, C5, C6, C7, C8 A1, A2, A3 I1	To be submitted: drug information sheet; patient information sheet about follow-up and treatment steps. Refer to the *Starting the Treatment* section of the patient information sheet. Staple a business card with the pharmacist’s name to the drug information sheet.
Management of side effects (about 15 days after first claim).	Identification and management of side effects. Revisit the expected treatment benefits and the benefits of continuing treatment. Importance of taking ADs as prescribed and the relationship between following treatment recommendations and side effects. Importance of persistence.	Side effects experienced and ways to manage them. Check if the patient has experienced an improvement in symptoms. Inquire if the patient is experiencing difficulties taking the drug as prescribed. Check the patient’s motivation for continuing treatment (despite the side effects). Verbal agreement for a follow-up at next renewal.	C9, C10 A2, A3 SE1, SE2, SE3 I2	Review the patient information sheet, especially the *Recognizing the side effects* section.
Monitoring treatment efficacy (at 30-day renewal).	Analysis of perceived treatment efficacy, mainly in relation to symptoms identified at the beginning of treatment. Identification and management of side effects, review those identified during the second consultation (15-day renewal). Treatment compliance. Treatment persistence.	Evaluation of treatment efficacy, benefits experienced. Side effects experienced and ways to manage them. Check treatment compliance. Check motivation and ability to continue taking medication (even if symptoms begin to improve).	C9 A4, A5, A6 SE1, SE2, SE3, SE4, SE5 I3	Review the patient information sheet, especially the *Assessing Early Benefits* section.
Assessment of treatment persistence (at 2 month renewal) *.	Treatment persistence. Analysis of perceived effectiveness, treatment benefits. Follow-up regarding management of side effects. Treatment compliance.	Check motivation and ability to continue taking medication (for the duration of treatment). Evaluation of effectiveness/benefits experienced. Side effects experienced and ways to manage them.	C9 A4, A5, A6 SE3, SE4, SE5 I3	Review the patient information sheet, especially the *Persistence* section.

* Repeat the fourth procedure at 6 months and every 6 months until the end of treatment.

**Table 5:** Matrix of objectives for intervention implementation (Step 5 of the Intervention Mapping protocol). Behavioral outcome: A drug therapy monitoring intervention of four brief consultations is systematically implemented for adult patients with MADs presenting with a new prescription for ADs at pharmacy.

Performance Objectives	Influencing Factors				
	Knowledge	Professional Identity/Attitude	Normative Beliefs	Self-Efficacy	Intention
PO1. The pharmacist becomes familiar with the content of the four consultations and adopts this systematic drug therapy monitoring intervention.	K1. The pharmacist knows the standards of practice related to drug therapy monitoring. K2. The pharmacist knows the objectives of systematic drug therapy monitoring. K3. The pharmacist knows the intervention strategies for this systematic drug therapy monitoring. K4. The pharmacist knows the content of the four brief interventions.	PI1. The pharmacist understands that this systematic drug therapy monitoring fits within his/her role as pharmacist. PI2. The pharmacist recognizes that it would be rewarding to implement this systematic drug therapy monitoring. A1. The pharmacist recognizes the patient benefits of implementing this drug therapy monitoring.	NB1. The pharmacist knows that the *Ordre des Pharmaciens du Québec* (Quebec Society of Pharmacists) is in favor of drug therapy monitoring NB2. The pharmacist believes that his/her colleagues would approve and encourage the implementation of systematic drug therapy monitoring.	SE1. The pharmacist feels able to identify patients initiating ADs for a MAD. SE2. The pharmacist feels able to inform the patient about the disease, the general mechanism of action of the treatment, treatment phases, onset of treatment efficacy, possible side effects and ways of dealing with them, complementary non-pharmacological measures to treat MADs, how to take the drug daily, and the importance of adherence to the medication for the duration of the prescription. SE3. The pharmacist feels able to question the patient’s intention to initiate the ADs and adhere to the treatment for the duration of the prescription. SE4. The pharmacist feels able to question the patient about the benefits he/she expects and experiences, and the presence of side effects and strategies to manage them. SE5. The pharmacist feels able to inform the patient about the potential risks of premature cessation.	I1. The pharmacist expresses a positive intention to implement systematic drug therapy monitoring, including four brief consultations in the pharmacy.
PO2. The pharmacist makes adjustments to his/her environment to facilitate the implementation of the intervention.		A2. The pharmacist recognizes that the implementation of the intervention is a team commitment. A3. The pharmacist recognizes the importance of holding consultations in a confidential area.		SE6. The pharmacist and pharmacy team agree on the strategies and tools they will use to perform and document the consultations. SE7. The pharmacist feels able to use these strategies and tools. SE8. The pharmacist has the required written information to give to the patient. SE9. The pharmacist identifies alternatives to employ when there are time constraints.	I2. The pharmacist expresses his/her intention to use these strategies and tools throughout drug treatment monitoring.
PO3. The pharmacist and pharmacy team agree on a time to implement the intervention.				SE10. The pharmacist feels able to implement the intervention at the chosen time.	I3. The pharmacist implements the intervention in pharmacy.

**Table 6:** Theoretical methods, application parameters, and practical applications for the implementation of the intervention (Step 5 of the Intervention Mapping protocol).

Methods (Related Theory)	Definition	Parameters	Practical Applications
Information about the Approval of Others (Theory of Planned Behavior) [51]	To provide information on what others think about a targeted behavior and whether others will approve or disapprove of the behavior.	People in the surrounding environment have positive expectations regarding the targeted behavior.	To encourage the person to be a role model.
Goal setting (Goal-Setting Theory) [54]	Prompting planning about what the person will do to achieve the behavioral goal.	Goals are difficult to achieve but attainable. People commit to achieving the goal.	General training in communication skills.
Guided practice (Social Cognitive Theory) [52]	Practice and repeat the behavior, discuss the experience, provide feedback.	Demonstration of particular skills is expected, requires the supervision of experienced people.	Demonstrate the expected behavior on video.
Facilitation (Social Cognitive Theory) [52]	Create an environment that facilitates action and reduces barriers to action.	Requires the identification of barriers and facilitators to action. Requires the power to make appropriate and real changes in the environment.	Restructuring the environment. Providing information on where and how to implement the intervention.
